# Negative Impact of Citral on Susceptibility of *Pseudomonas aeruginosa* to Antibiotics

**DOI:** 10.3389/fmicb.2021.709838

**Published:** 2021-07-05

**Authors:** Alexandre Tetard, Sarah Foley, Gaëtan L. A. Mislin, Jean-Michel Brunel, Estefania Oliva, Freddy Torrealba Anzola, Andy Zedet, Bruno Cardey, Yann Pellequer, Christophe Ramseyer, Patrick Plésiat, Catherine Llanes

**Affiliations:** ^1^UMR CNRS 6249 Chrono-Environnement, Université Bourgogne Franche-Comté, Besançon, France; ^2^CNRS/Université de Strasbourg UMR 7242 Biotechnologie et Signalisation Cellulaire, Illkirch, France; ^3^UMR_MD1, U-1261, Aix Marseille Université, INSERM, SSA, MCT, Marseille, France; ^4^Plateforme d’Analyse Chimique de Strasbourg-Illkirch (PACSI), Faculté de Pharmacie de Strasbourg, Illkirch, France; ^5^PEPITE EA4267, Université de Bourgogne Franche-Comté, Besançon, France; ^6^Centre National de Référence de la Résistance aux Antibiotiques, Centre Hospitalier Universitaire de Besançon, Besançon, France

**Keywords:** *Pseudomonas aeruginosa*, essential oils, citral, antibiotic resistance, efflux, tobramycin-citral Schiff base, colistin-citral Schiff base

## Abstract

Essential oils (EOs) or their components are widely used by inhalation or nebulization to fight mild respiratory bacterial infections. However, their interaction with antibiotics is poorly known. In this study we evaluated the effects of citral, the main component of lemongrass oil, on *in vitro* susceptibility of *Pseudomonas aeruginosa* to antibiotics. Exposure of strain PA14 to subinhibitory concentrations of citral increased expression of operons encoding the multidrug efflux systems MexEF-OprN and MexXY/OprM, and bacterial resistance to anti-pseudomonal antibiotics including imipenem (twofold), gentamicin (eightfold), tobramycin (eightfold), ciprofloxacin (twofold), and colistin (≥128-fold). Use of pump deletion mutants showed that in addition to efflux other mechanisms were involved in this citral-induced phenotype. Determination of Zeta potential suggested that citral impairs the cell surface binding of aminoglycosides and colistin used at low concentrations (≤10 μg/mL). Moreover, experiments based on Raman spectroscopy and high-resolution mass spectrometry demonstrated formation of a Schiff base between the aldehyde group of citral and amino-groups of tobramycin and colistin. Chemical synthesis of tobracitryl, the imine compound resulting from condensation of citral and tobramycin, confirmed the loss of antibiotic activity due to adduct formation. Altogether these data point to the potential risk concern of self-medication with EOs containing citral in patients suffering from *P. aeruginosa* chronic lung infections and being treated with aerosols of aminoglycoside or colistin.

## Introduction

Essential oils (EOs) are complex mixtures of volatile compounds, produced by plants. Some possess antimicrobial activities, and are thus commonly used as self-medication to treat mild respiratory infections (Inouye et al., 2001). Noteworthily, an increasing number of studies suggest that EOs could be useful as adjunctive therapy in cystic fibrosis (CF) patients chronically infected with *Haemophilus influenzae* (Balazs et al., 2019), *Staphylococcus aureus, Stenotrophomonas maltophilia, Achromobacter xylosoxidans* (Pesavento et al., 2016), or *Pseudomonas aeruginosa* (Ragno et al., 2020). For instance, extracts of bay, cinnamon, clove, pimento, thyme, rosemary, oregano and lemongrass have been reported to inhibit *in vitro* the growth of *P. aeruginosa*, a major pathogen in CF lung (Hammer et al., 1999; Swamy et al., 2016). However, clinical evidence that such EOs or their components would have the same efficacy *in vivo* is lacking.

The main constituent of lemongrass oil, citral, is actually a mixture of two isomeric acyclic monoterpene aldehydes (C_1__0_H_1__6_O): geranial (*trans*-citral or citral A), and neral (*cis*-citral or citral B). This common flavor compound occurs at various concentrations in *Citrus* oil, as well as in leaves and fruits of myrtle trees, basil, lemon, lime, lemongrass, orange, and bergamot. Despite its moderate volatility, citral exhibits an interesting antipseudomonal activity by gaseous contact, close to that of cinnamaldehyde, another aldehyde present in cinnamon bark oil (Inouye et al., 2001). However, when in solution, citral appears to be more active on Gram positive bacteria (MIC of 0.1 mg/mL for *Staphylococcus aureus*) than on Gram negatives (MIC ≥ 0.5 mg/mL) (Hyldgaard et al., 2012; Shi et al., 2016; Gupta et al., 2017; Thielmann and Muranyi, 2019). In the foodborne pathogen *Cronobacter sakasakii*, this aldehyde triggers pleiotropic effects including a shift in ATP concentration, acidification of the cytosol, cell membrane hyperpolarization, and damages to the cell wall, that would result in or contribute to cell death (Shi et al., 2016). Beside these alterations, citral is able to lower the production of virulence factors in *Vibrio parahaemolyticus* (Sun et al., 2019) and *C. sakazakii* (Shi et al., 2017), as well as to prevent the synthesis of quorum sensing auto-inducer *N*-dodecanoyl-L-homoserine lactone in *Pseudomonas putida* (Jaramillo-Colorado et al., 2012). Because of these different properties, citral might have potential applications in the treatment of some bacterial infections (Shah et al., 2011).

In the context of CF, lemongrass oil (that contains about 65–85% citral) was found to inhibit the *in vitro* growth of drug resistant strains belonging to the *Burkholderia cepacia* complex (Vasireddy et al., 2018). On *P. aeruginosa*, its effects ranged from negligible (MIC > 17 mg/mL) (Cox and Markham, 2007) to mild (MIC = ∼ 9 mg/mL) (Hammer et al., 1999). Other EOs known to contain citral (lemon, bergamot, orange, myrtle, and basil oils) yielded similar results on CF or reference strains (Ragno et al., 2020). Likely accounting for this poor activity, *P. aeruginosa* together with other *Pseudomonas* sp. such as *P. citronellolis* and *P. mendocina*, is able to degrade citral and to use it as sole source of carbon and energy (Cantwell et al., 1978; Hoschle and Jendrossek, 2005). In addition, recent studies demonstrated that *P. aeruginosa* copes with the toxic effects of EOs by overproducing RND efflux pumps, a family of polyspecific transporters that plays a major role in both natural and acquired resistances to antibiotics (Papadopoulos et al., 2008; Pesingi et al., 2019; Tetard et al., 2019). For example, we found that subinhibitory concentrations of cinnamaldehyde (from cinnamon bark oil) result in an electrophilic stress to which *P. aeruginosa* adapts by upregulating not less than four RND pumps: MexAB-OprM, MexCD-OprJ, MexEF-OprN, and MexXY/OprM. This general increase in cell efflux activity is associated with a higher resistance of *P. aeruginosa* to ß-lactams (via MexAB-OprM), aminoglycosides (via MexXY/OprM), and fluoroquinolones (all systems) (Tetard et al., 2019). Activation of the *mexXY* and *mexEF-oprN* operons was lost when cinnamaldehyde was substituted in the culture medium with its catabolite cinnamic alcohol, a result that highlights the role of aldehyde group in cell stress.

In this work, we studied the impact of the acyclic aldehyde citral on the susceptibility of *P. aeruginosa* to several antibiotics commonly prescribed to treat CF lung infections. While citral has a limited antimicrobial activity on *P. aeruginosa* by itself, it may have antagonistic effects when combined with antibiotics. An efflux-independent mechanism is proposed to explain some of these adverse effects.

## Results and Discussion

### *P. aeruginosa* Is Highly Resistant to Citral

**FIGURE 1:**
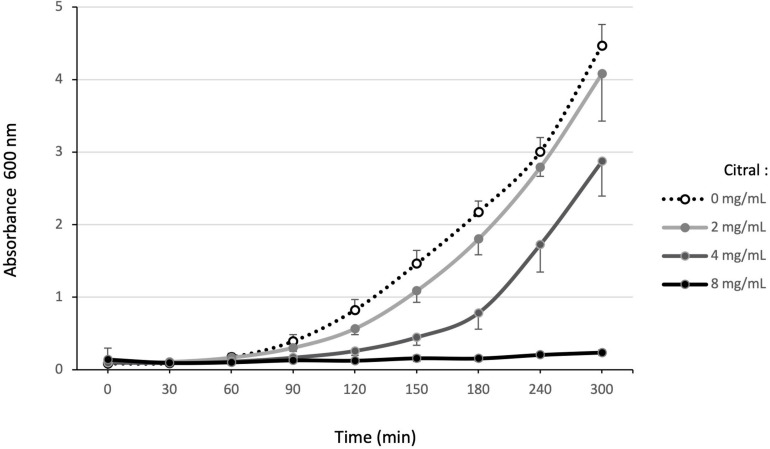
Effect of increasing citral concentrations on *P. aeruginosa* reference strain PA14. Bacterial growth in MHB supplemented with different concentrations of citral (0, 1, 2, 4, and 8 mg/mL) was monitored at *A*_60__0__*n*__*m*_ over 5 h. The results presented are mean values, calculated from three independent bacterial cultures.

Susceptibility of reference (PA14, PAO1, LESB58) and environmental strains (1341, 1393, 1423) was first assayed by the disk diffusion method (Supplementary Figure 1). As no inhibition zone appeared around disks loaded with 5 μL (4.44 mg) citral, we monitored the growth of PA14 exposed to increasing concentrations of the compound. After 5 h, no visible growth developed in presence of 8 mg/mL citral, while lower concentrations (4 or 2 mg/mL) slowed down the bacterial multiplication (Figure 1). After 18 h, only the culture exposed to 16 mg/mL remained negative (data not shown). In comparison, the MIC of cinnamaldehyde was more than 20-fold lower (0.7 mg/mL) for PA14, confirming the weak toxicity of citral on *P. aeruginosa*.

### Citral Induces Efflux Pumps Expression and Antibiotic Resistance in *P. aeruginosa*

**FIGURE 2:**
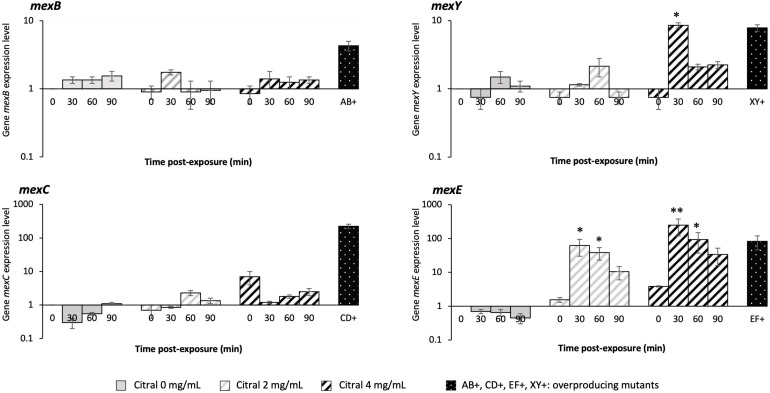
Relative expression levels of *P. aeruginosa* genes *mexB, mexC, mexE*, and *mexY*. Gene transcripts were measured over time by RT-qPCR in strain PA14 exposed to 0, 2, and 4 mg/mL citral. The genes were considered as activated (single asterisk) when their expression was ≥ 3-fold (*mexB*), ≥ 5-fold (*mexY*), and ≥ 20-fold (*mexE* and *mexC*) that of unexposed PA14 at *t_0_.* A double asterisk indicates when mRNA amounts were higher than those of efflux overproducing mutants PA14*mexAB*^+^ (*mexB*: 3.6-fold that of PA14), PA14*mexCD*^+^ (*mexC*: 257-fold), PA14*mexEF*^+^ (*mexF*: 47-fold), and PA14*mexXY*^+^ (*mexY:* 8.7-fold), derived from parental strain PA14. Values are means of two independent experiments each including two technical replicates.

**FIGURE 3:**
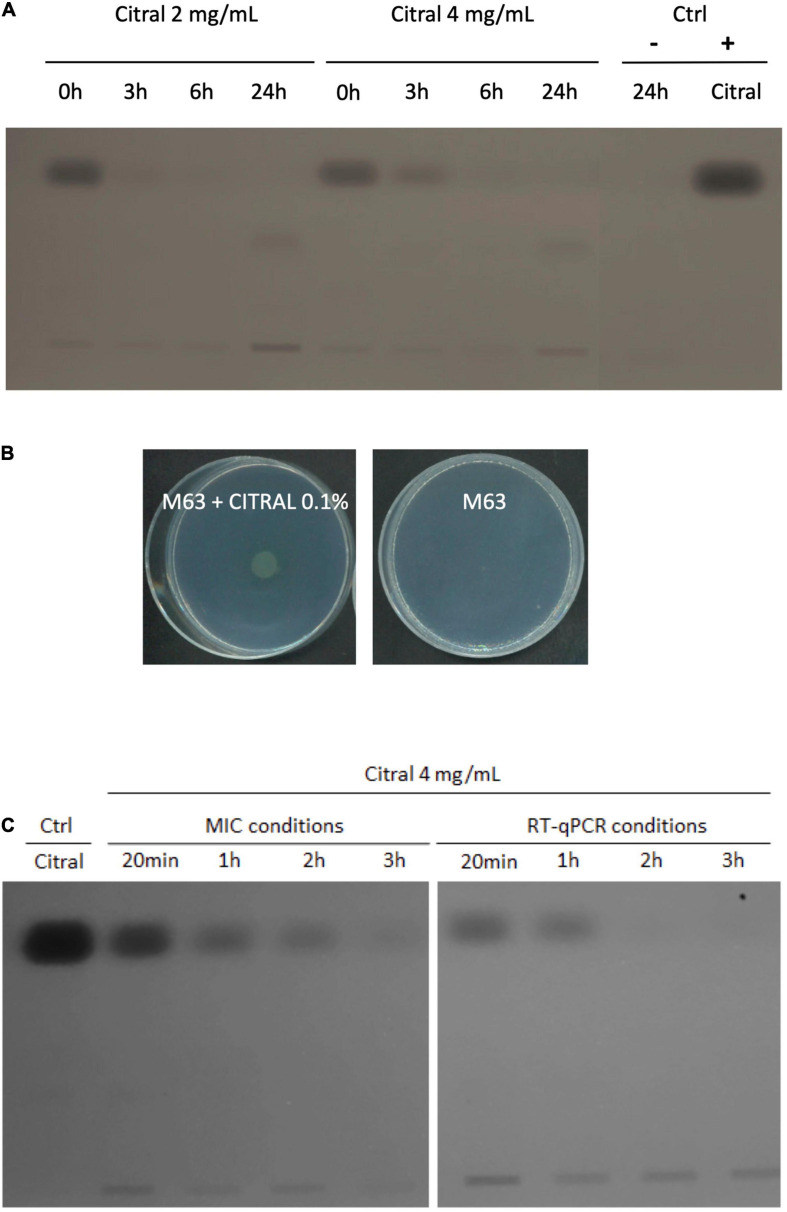
Degradation of citral by *P. aeruginosa* strain PA14. **(A)** TLC analysis of culture supernatants of strain PA14 after 0, 3, 6, and 24 h of incubation with 2 or 4 mg/mL citral. Ctrl: supernatant of PA14 growth medium without citral after 24 h (–), citral control sample 5 μg (+). **(B)** Growth of PA14 on minimal medium M63 with or without citral as sole carbon source (after 48 h at 37°C). **(C)** TLC analysis of citral consumption by using an initial inoculum of 5.10^5^ CFU/mL (MICs conditions) or 1.5.10^8^ CFU/mL (RT-qPCR conditions) in MHB.

Exposure of *P. aeruginosa* to some EO components (such as cinnamaldehyde), triggers a complex adaptive response that in part relies on a fast increase in efflux activity (Tetard et al., 2019). We thus assessed the impact of citral on the expression of four RND efflux operons known to mediate antibiotic resistance in this species, namely *mexAB-oprM*, *mexCD-oprJ*, *mexEF-oprN*, and *mexXY* (Li et al., 2015). Relative expression levels of representative genes *mexB, mexC, mexE*, and *mexY* were determined in strain PA14 by real-time quantitative PCR (RT-qPCR) at different time points of citral exposure. As shown in Figure 2, *mexE* and *mexY* were activated in the bacteria challenged with sub-minimal inhibitory concentrations (subMICs) of citral (2 and 4 mg/mL). Their expression peaked rapidly (30 min) and then dropped (60 min) below the threshold level. Likely accounting for this transient gene induction, citral was rapidly degraded by strain PA14 after 1 h of incubation (Figure 3A), as it is consumed by the bacterium as source of carbon and energy (Figure 3B). In contrast, a weak impact on *mexB* and *mexC* activity was documented in cultures with citral, these genes being overexpressed 2.5- and 31-fold less than the levels of control mutants constitutively overexpressing the corresponding pumps, respectively.

**FIGURE 4:**
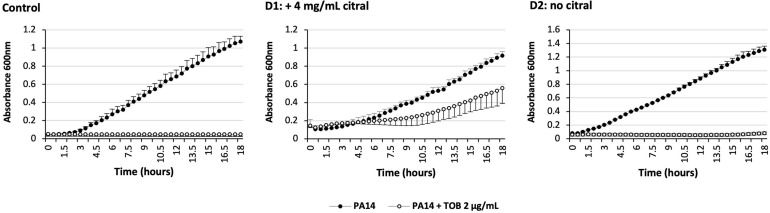
Impact over time of citral on *P. aeruginosa* susceptibility to tobramycin. Control: PA14 was grown in MHB with (open circle) or without (solid circle) 2 μg/mL tobramycin (TOB). **Day 1 (D1)**: PA14 was grown in MHB containing 4 mg/mL citral and complemented or not with TOB. **Day 2 (D2)**: Tobramycin tolerant cells of D1 (open circle) were grown in a fresh medium without citral, and complemented or not with TOB. The results are presented as mean values calculated from three independent bacterial cultures.

Because overproduction of MexEF-OprN and MexXY/OprM results in an increased drug resistance in *P. aeruginosa* (Llanes et al., 2011; Guenard et al., 2014), we determined the susceptibility of strain PA14 to various antibiotics in the presence and absence of citral, by the microdilution method (Table 1). Consistent with operon *mexEF-oprN* being overexpressed, MICs of ciprofloxacin and chloramphenicol increased 2- and >32-fold, respectively upon citral exposure (2 or 4 mg/mL). Resistance of PA14 also increased twofold to imipenem, while it decreased twofold to ticarcillin, likely because of inverse coregulation of MexEF-OprN with carbapenem-specific porin OprD and pump MexAB-OprM, respectively (Maseda et al., 2004; Sobel et al., 2005; Table 1). In addition, MICs of aminoglycosides (tobramycin and gentamicin), which are exported by MexXY/OprM, augmented up to eightfold in cultures exposed to 4 mg/mL citral. Supporting the notion of reversible adaptive response to the presence of the aldehyde, PA14 cells recovered their wild-type susceptibility phenotype when subcultured in citral-free medium (for example, to tobramycin, Figure 4). It should be noted that while RT-qPCR experiments demonstrated a relatively brief induction of operons *mexEF-oprN* and *mexXY* by citral (about 1 h), the impact of the aldehyde on antibiotic resistance (MICs) persisted much longer (18 h post-exposure). Because higher bacterial densities (1.5 × 10^8^ CFU/mL) are needed for gene expression assays than in MIC experiments (5 × 10^5^ CFU/mL), citral is rapidly metabolized under the RT-qPCR culture conditions, resulting in rapid decline of the stressor in the growth medium. In contrast, its presence in drug susceptibility testing conditions was still detected by thin layer chromatography (TLC) after 3 h of incubation (Figure 3C).

**TABLE 1:** Influence of citral on *P. aeruginosa* susceptibility to antibiotics.

Antibiotics^*a*^	Citral (mg/mL)		MICs of antibiotics (μg/mL) of strains^*b*^				
		PA14	PA14ΔXY	PA14*mexXY*^+^	PA14ΔEFN	PA14*mexEFN*^+^	PA14ΔXY/EFN
Ticarcillin	**0**	32			32	16	
	**0.5**	32			32	16	
	**1**	32			32	16	
	**2**	*16*			32	16	
	**4**	*16*			32	16	
Imipenem	**0**	0.5				4	
	**0.5**	0.5				4	
	**1**	0.5				4	
	**2**	**1**				4	
	**4**	**1**				4	
Gentamicin	**0**	1	0.25	4			
	**0.5**	1	0.25	4			
	**1**	**2**	0.25	4			
	**2**	**4**	**0.5**	**8**			
	**4**	**8**	**1**	**8**			
Tobramycin	**0**	0.5	0.25	2			
	**0.5**	0.5	0.25	2			
	**1**	**1**	0.25	2			
	**2**	**2**	**0.5**	**4**			
	**4**	**4**	**1**	**4**			
Ciprofloxacin	**0**	0.125	0.125	0.25	0.125	2	0.06
	**0.5**	0.125	0.125	0.25	0.125	2	0.06
	**1**	0.125	0.125	0.25	0.125	2	0.06
	**2**	**0.25**	**0.25**	0.25	0.125	2	0.06
	**4**	**0.25**	**0.5**	**0.5**	0.125	2	0.06
Chloramphenicol	**0**	64	64		64	1,024	
	**0.5**	**128**	**128**		64	1,024	
	**1**	**256**	**256**		64	1,024	
	**2**	**256**	**256**		64	1,024	
	**4**	**512**	**512**		64	1,024	
Colistin	**0**	0.5	0.5				
	**0.5**	**1**	**1**				
	**1**	**2**	**4**				
	**2**	**8**	**8**				
	**4**	** ≥64**	**≥64**				

*^*a*^Ticarcillin, is substrate of MexAB-OprM; gentamicin and tobramycin are substrates of MexXY/OprM; ciprofloxacin is a substrate of MexAB-OprM, MexEF-OprN and MexXY/OprM; chloramphenicol is substrate of MexAB-OprM and MexEF-OprN; porin OprD, which is inversely co-regulated with MexEF-OprN, is the main uptake pathway of imipenem; colistin is substrate of MexXY/OprM in the specific case of PmrAB mutants. ^*b*^When increased (decreased) by the presence of citral, MIC values are indicated in bold (italic). All the experiments were made in triplicate. Blank spaces with no MIC data denote that no drug susceptibility testing was conducted as they are not relevant.*

### Efflux Is Not the Only Mechanism Interfering With Antibiotic Activity

To better assess the role of efflux pumps in citral-promoted resistance to antibiotics, we used PA14 mutants harboring single or dual deletions of operons *mexEF-oprN* and *mexXY* (PA14ΔEFN, PA14ΔXY, and PA14ΔEFN/XY, respectively) (Table 1). As expected, inactivation of *mexEF-oprN* completely abolished the development of ciprofloxacin resistance in mutant PA14ΔEFN exposed to citral. On the other hand, deletion of *mexXY* had a more limited impact on aminoglycosides MIC variations (compare PA14 with PA14ΔXY) and virtually no effect on colistin resistance, suggesting that additional causes contribute to the loss antibiotic activity under citral exposure (Table 1).

### Citral Reduces Cell Surface Binding of Aminoglycosides and Colistin at Low Antibiotic Concentrations

**FIGURE 5:**
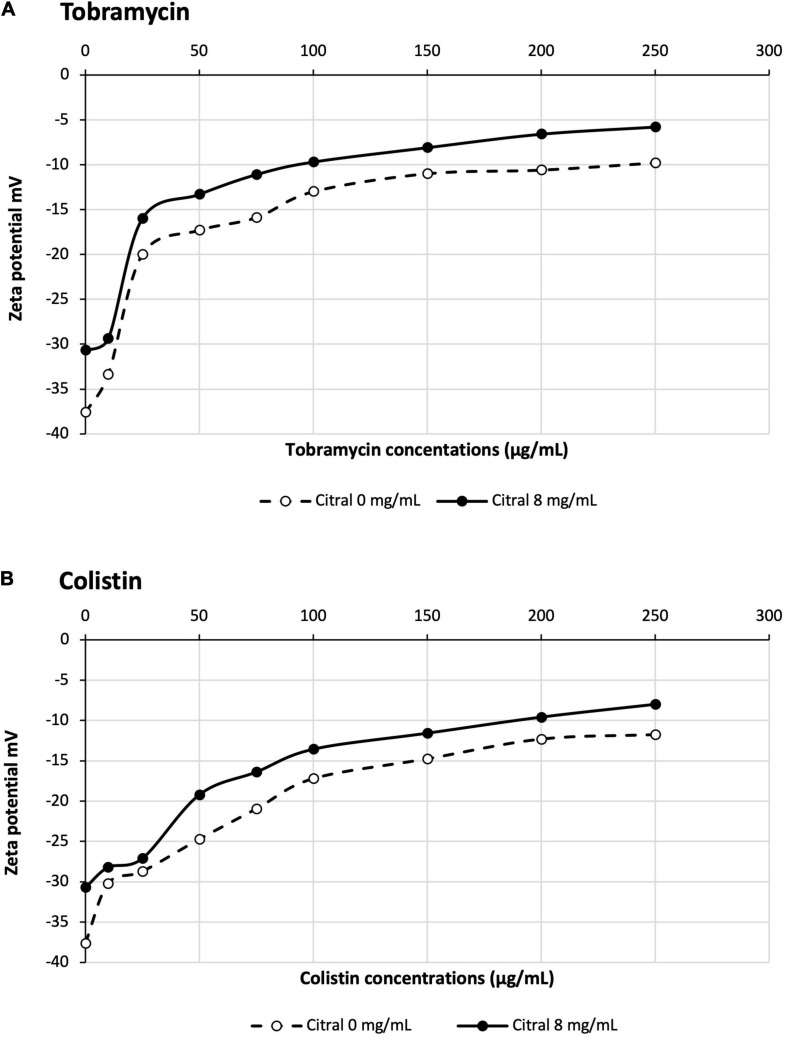
Impact of citral on Zeta potential of PA14 cells treated with tobramycin **(A)** or colistin **(B)**.

Aminoglycosides and colistin are polycationic molecules at neutral pH. The electrostatic interaction of their amine groups with the phosphate residues of lipopolysaccharides (LPS) is believed to promote the penetration of these drugs across the outer membrane, via a mechanism called “self-uptake pathway” that does not require the presence of porins (Krause et al., 2016; O’Driscoll et al., 2018). We wondered whether citral would reduce the net negative charges of bacterial surface, and thus the passage of aminoglycosides and colistin into the cell interior. To check this hypothesis, we measured the Zeta potential of strain PA14 before and after citral treatment. As previously reported (Tashiro et al., 2010), untreated PA14 cells exhibited a negative surface charge of −38 mV. This value slightly raised to −30 mV when the bacteria were preincubated with citral, suggesting that a number of outer membrane polar groups are masked by this very hydrophobic molecule when it partitions into the lipid bilayer. In absence of citral, the potential yielded a value of −10 and −12 mV with increasing concentrations of colistin and tobramycin, respectively in agreement with the notion that these antibiotics interact with and neutralize the negatively charged groups of LPS (Figure 5). Interestingly, citral reduced the magnitude of variation in Zeta potential when the bacterial cells were exposed to low concentrations of polycations (10 μg/mL) (Figure 5). A plausible explanation for these results is that citral impairs the binding and penetration of amine antibiotics, at least under certain experimental conditions. The further discovery that citral antagonizes polycationic antibiotics not only in *P. aeruginosa* but also in *P. putida, Acinetobacter baumannii* and *Escherichia coli* prompted us to search other origins to its negative effects (Supplementary Figure 2).

### Citral Forms an Adduct With Tobramycin

**FIGURE 6:**
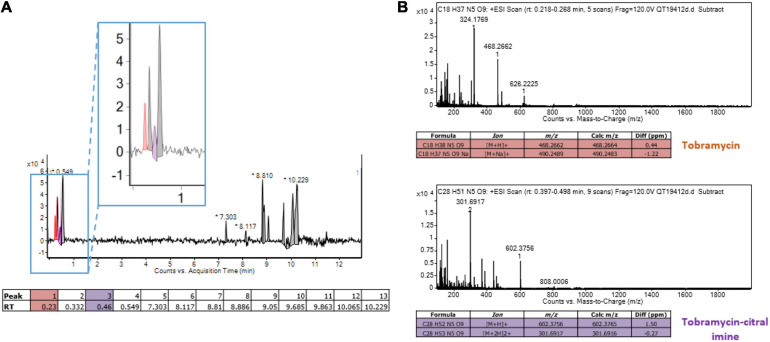
Detection of a monoadduct of citral-tobramycin. **(A)** Liquid chromatography of a mixture tobramycin and citral (ratio 1/600). The peak corresponding to tobramycin is colored in orange and the peak corresponding to the tobramycin-citral imine is colored in purple. **(B)** Mass spectrometry analysis (Electrospray in positive mode) of tobramycin and tobramycin-citral imine from liquid chromatography. The target ions/formulas are displayed in the tables.

**FIGURE 7:**
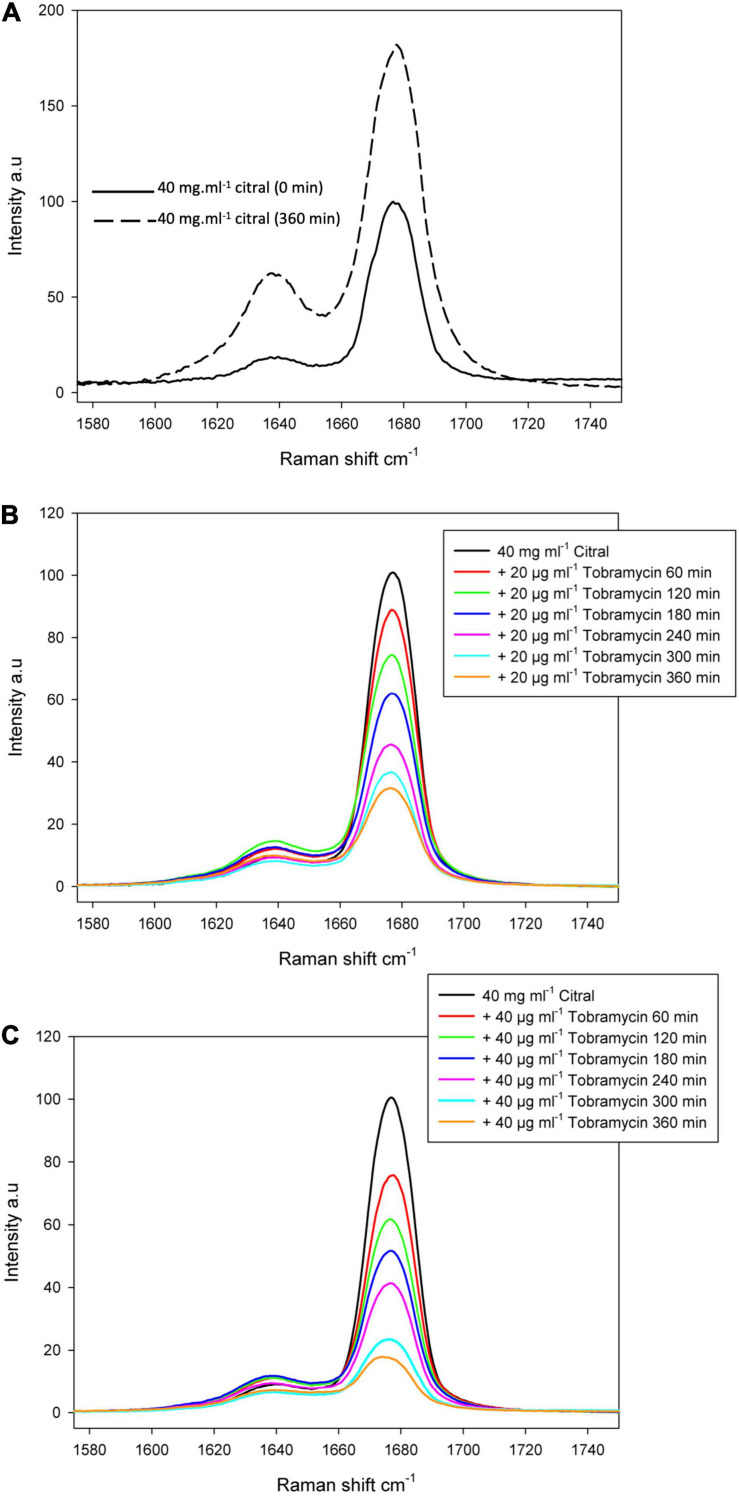
Raman spectrum of the citral C=O band. Raman spectrum of the citral C=O band was centered at 1676 cm^– 1^ and measured before and after 360 min at 37°C, to confirm citral can be still detected after this incubation time **(A)**. The reaction between citral and tobramycin was followed over the same period of 6 h by adding 20 μg/mL **(B)** or 40 μg/mL of tobramycin **(C)**.

Reactive amino groups of aminoglycosides and polymyxins are subject to react with the aldehyde groups of other compounds, to form Schiff bases (RC=N-) (Fan et al., 2019). In this context, the more reactive amine group appears to be that in position 6’ of the nebrosamin part of tobramycin (Alkhzem et al., 2020). If so, formation of some adducts could impair the activity of these antibiotics, by preventing their entry into bacterial cells and/or by inhibiting their binding to specific cellular targets. In order to demonstrate the formation of such imine conjugates, we carried out LC-HRMS (electrospray) experiments on mixtures of tobramycin/citral (ratio 1/600) and of colistin/citral (1/1500). This approach allowed the detection of a mono-imine tobramycin-citral derivative (calculated mass for [M + H]^+^ C_2__8_H_5__2_N_5_O_9_: 602.3765; measured: 602.3756) along with free tobramycin (Figure 6), but failed to evidence formation of a similar compound between colistin and citral. To prevent a possible hydrolysis of imine during the preparation of the MS sample, a prior reduction of the colistin/citral mixture using sodium cyanoborohydride was performed, but the corresponding reduced colistin-citral adduct was not detected by LC-HRMS (data not shown). The loss of citral C=O chemical bond (in favor of C=N) in presence of tobramycin or colistin was then assessed by Raman spectroscopy at 1676 cm^–1^ (Figure 7). In control experiments, we could observe that after 6 h of incubation at 37°C (Figure 7A), the C=O vibration increased in intensity, probably due to an improved solubilization of citral in water. At the opposite, the addition of either 20 or 40 μg/mL of tobramycin resulted in a decrease of C=O band intensity (proportional with tobramycin concentration) over the incubation time (Figures 7B,C). A similar result was obtained with 40 μg/mL colistin, but not with ticarcillin, an antibiotic molecule devoid of amine group (Supplementary Figures 3A,B).

To objectivate the formation of inactive citral-based adducts over time, we compared the susceptibility of strain PA14 to a selection of antibiotics previously preincubated with or without citral. As indicated in Table 2, MICs of gentamicin, tobramycin, and colistin gradually increased with the preincubation time, up to 32-fold after 24 h of contact, while the activity of ticarcillin remained constant under these conditions.

**TABLE 2:** Preincubation with citral reduces the activity of aminoglycosides and colistin on *P. aeruginosa* strain PA14.

Antibiotics ^*a*^	Citral (mg/mL)	MICs of antibiotics (μg/mL) after preincubation with citral			
		0 h	3 h	6 h	24 h
Gentamicin	**0**	1	1	1	1
	**0.5**	1	1	**2**	**4**
	**1**	**2**	**2**	**4**	**16**
Tobramycin	**0**	0.5	0.5	0.5	0.5
	**0.5**	0.5	**2**	**2**	**4**
	**1**	**1**	**2**	**4**	**16**
Colistin	**0**	0.5	0.5	0.5	0.5
	**0.5**	**1**	**4**	**4**	**16**
	**1**	**2**	**4**	**8**	**16**
Ticarcillin	**0**	32	32	32	32
	**0.5**	32	32	32	32
	**1**	32	32	32	32

*When increased by citral, antibiotic MIC values are indicated in bold; when further increased by preincubation, MICs are underlined. Ticarcillin was used as a negative control since its does not interact with citral. All the experiments were made in triplicate.*

**FIGURE 8:**
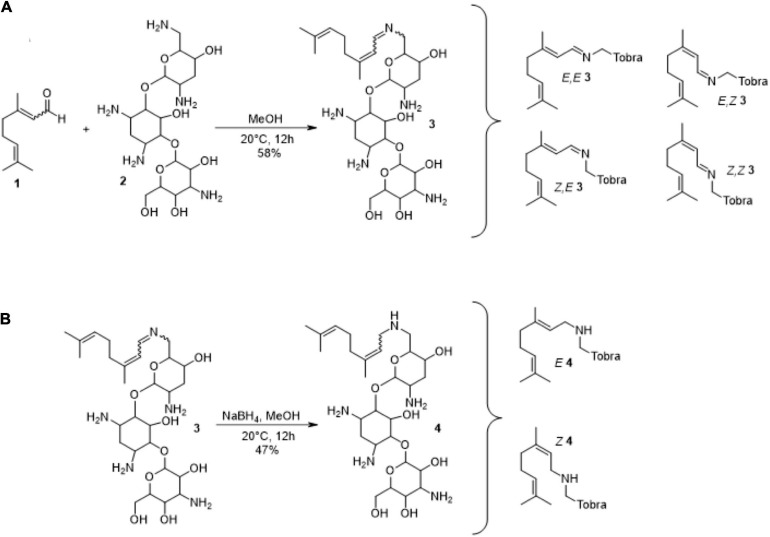
Synthesis of tobracitryl resulting from the condensation of citral with tobramycin. **(A)** Synthesis of tobracitryl **3** (mixture of four isomers), **(B)** Reduction of tobracitryl **3** in tobracitryl **4** (mixture of two isomers). The proposed structures are the most probable according to the fact that the amine group N-6’ is the most reactive due to its highest pKa (Alkhzem et al., 2020).

The lack of antibacterial activity of imine adducts was finally tested by using tobracitryl, a product resulting from the condensation of citral with tobramycin in a 1:1 ratio as a mixture of four isomers in 58% yield (Figure 8A). Interestingly, MIC of tobracitryl for PA14 was 16-fold higher than that of tobramycin (8 vs. 0.5 μg/mL). However, as the imine appeared to be unstable in water, re-forming spontaneously the initial amine, we synthesized a stable tobracitryl derivative, by reduction of the imine group of tobracitryl into an amine one in 47% yield (Figure 8B). The activity of this stable adduct proved to be very low (MIC >64 μg/mL), confirming our hypothesis that citral is able to spontaneously inactivate amine antibiotics by forming Schiff bases.

## Conclusion

In the global context of increasing antibiotic resistance, therapeutic indications of EOs need to be reassessed. Because they are obtained from plants, these natural products are often viewed as a safe and affordable alternative to antibiotics to treat mild respiratory infections mostly due to Gram positive bacterial species (Inouye et al., 2001). However, their activity is several orders of magnitude lower than that of antibiotics, their MICs being around 1–8 mg/mL. Moreover, data supporting their clinical efficacy are scarce and their toxicity is often overlooked (Calhoun et al., 2019). As a matter of fact, the Cystic Fibrosis Foundation (CFF) warns against using EOs by nebulization as this may cause lung inflammation^1^. Based on *in vitro* experiments, a number of studies suggest that EOs could be used to synergize antibiotics against multidrug resistant Gram negative bacteria (for a review Langeveld et al., 2014). While their conclusions still need to be confirmed in clinical situations, data accumulate showing that some EO components show adverse effects *in vitro* on major antibiotics (Van Vuuren et al., 2009; Moussaoui and Alaoui, 2016). For example, compounds with aldehyde groups such as cinnamaldehyde and citral induce an electrophilic stress response in *P. aeruginosa* implying multidrug efflux transporters such as MexEF-OprN and MexXY/OprM, which in turn increases the resistance of the bacterium to pump-substrate antibiotics (Tetard et al., 2019). Similar effects on the pathogen are obtained with glutaraldehyde, a biocide used for cleaning and disinfection in hospitals, and malondialdehyde, a catabolite of polyunsaturated fatty acid peroxidation found in CF lungs (data not shown).

The present work reveals the potential risk of using citral with aminoglycosides or polymyxins to treat human infections. Since these antibiotics are essentially administered by intravenous route to combat hard-to-treat nosocomial infections, their coadministration with citral is not well justified in view of the very weak efficacy of the product on Gram negative species, and absence of validated protocols. In contrast, in the context of CF and chronic obstructive pulmonary disease (COPD), aerosols of tobramycin and colistin are administered iteratively as maintenance therapy to control lung colonization with *P. aeruginosa.* Whether self-medication with EO formulations containing citral can be detrimental to these therapeutic regimens is unknown. Data are lacking on the concentrations of EO components achieved in airways after nebulization, inhalation or ingestion. Our results tend to demonstrate that aerosolization of antibiotics together with some volatile antimicrobials yielding 1:8 MIC locally *in vivo* may result in antagonistic interactions due to bacteria-dependent mechanisms (e.g., increased efflux, lower outer membrane permeability) combined with chemical inhibition of antibiotic active groups. Thus, patients with CF or COPD should be informed about the risk of self-medication with EOs containing citral or cinnamaldehyde, as these off the shelf supplements are distributed without restriction, and are usually considered as non-toxic.

## Materials and Methods

### Bacterial Strains, Plasmids and Growth Conditions

The bacterial strains and plasmids used in this study are listed in Table 3. All the bacterial cultures were incubated at 37°C in Mueller-Hinton broth (MHB) provided with adjusted concentrations of Ca^2+^ (from 20 to 25 μg/mL) and Mg^2+^ (from 10 to 12.5 μg/mL) (Becton Dickinson and Company, Cockeysville, MD, United States), or on Mueller-Hinton agar cation-adjusted (MHA) (Bio-Rad, Marnes-la-Coquette, France) supplemented with antibiotics when required, or on minimal medium M63 with 0.1% citral as carbon and energy source (Ausubel et al., 2000). Citral, a mixture of citral B and citral A (1:2) (Sigma-Aldrich, Saint Quentin Fallavier, France) was dissolved in 1:6 (vol:vol) aqueous solution of ethanol before use (stock solution 148 mg/mL).

**TABLE 3:** Strains and plasmids used in the study.

Stains	Description	Origin
***Pseudomonas aeruginosa***		
PA14	Wild type reference strain, susceptible to antibiotics	Liberati et al., 2006
PAO1	Wild type reference strain, susceptible to antibiotics	B. Holloway
LESB58	Clinical strain isolated from cystic fibrosis patient (United Kingdom)	C. Winstanley
1341	Environmental strain isolated from surface water (France)	This study
1393	Environmental strain isolated from surface water (France)	This study
1423	Environmental strain isolated from surface water (France)	This study
PA14ΔEFN	PA14 mutant lacking operon *mexEF-oprN*	Tetard et al., 2019
PA14*mexEF*^+^	MexEF-OprN overproducing mutant derived from PA14	Tetard et al., 2019
PA14ΔXY	PA14 mutant lacking operon *mexXY*	Tetard et al., 2019
PA14*mexXY*^+^	MexXY/OprM overproducing mutant derived from PA14	Tetard et al., 2019
PA14ΔEFNΔXY	PA14 mutant lacking operons *mexEF-oprN* and *mexXY*	Tetard et al., 2019
**Other bacterial species**		
ATCC 25922	*Escherichia coli* reference strain	Minogue et al., 2014
CIP 70.10	*Acinetobacter baumannii* reference strain	Marchand et al., 2004
KT2440	*Pseudomonas putida* reference strain	Nelson et al., 2002
SA1	Clinical strain of *Staphylococcus aureus* isolated at the Besançon teaching hospital (France)	This study

### Drug Susceptibility Testing

Citral susceptibility of *P. aeruginosa* strains was assessed on MHA. Paper disks loaded with 4.44 mg (5 μL) citral were deposited onto the surface of seeded MHA plates. The size of the inhibition zone was measured after 18 h of incubation at 37°C ± 1.

The minimum inhibitory concentrations (MICs) of selected antibiotics were determined by the standard serial twofold microdilution method, as recommended by the CLSI (Clinical and Laboratory Standards Institute, 2020). Briefly, log-phase bacterial cultures adjusted at 10^5^ CFU/mL were distributed in 96-well sterile microplates by using a Freedom EVO^®^ platform (Tecan, Männedorf, Switzerland), were incubated at 37°C ± 1, and then examined visually after 18 h of growth. All the MIC experiments were performed in triplicate. The impact of citral on antibiotic MICs was assessed at different subinhibitory concentrations (0.5, 1, 2, or 4 mg/mL). A negative control with 2% ethanol was made in parallel.

Chemical interaction of citral with amine antibiotics gentamicin, tobramycin, and colistin was studied by incubating each antibiotic with 0, 0.5, or 1 mg/mL citral at 37°C for 0, 3, 6, or 18 h in MHB, before performing MIC experiments (see above). Serial twofold dilutions of the antibiotic solutions with and without citral were added to 10^5^ CFU, and incubated at 37°C ± 1 for 18 h. A negative control was made in parallel with the non-amine antibiotic ticarcillin. A similar assay was carried out with paper disks impregnated with tobramycin pre-incubated or not with citral, and deposited onto the surface of MHA plates inoculated with a strain of *S. aureus* (Table 3).

### RT-qPCR Experiments

Specific gene expression levels were measured by quantitative PCR after reverse transcription (RT-qPCR), as previously described (Richardot et al., 2016). RNA was reverse transcribed with ImProm-II reverse transcriptase, according to the manufacturer’s protocol (Promega, Madison, WI, United States). Amounts of specific cDNA were assessed on a Rotor Gene RG6000 instrument (Qiagen, Courtaboeuf, France) by using the QuantiFast SYBR green PCR Kit (Qiagen) and primers annealing to target gene sequences (Supplementary Table 1). For each strain, target gene mRNA levels were normalized to that of housekeeping gene *rpsL*, and were expressed as a ratio to the transcript levels of strain PA14. Mean gene expression values were calculated from two independent bacterial cultures, each assayed in duplicate. As shown previously, transcript levels of *mexB* ≥ 3-fold, *mexY* ≥ 5-fold, *mexC*, and *mexE* ≥ 20-fold those of PA14, were considered as significantly increased because associated with a ≥ 2-fold higher resistance to respective pump substrates (Llanes et al., 2013; Richardot et al., 2016).

### Metabolite Extraction and Detection Using Thin Layer Chromatography (TLC)

A standard working solution of citral was prepared by diluting aliquots of >98% stock solutions in methanol. Overnight bacterial cultures were diluted into 25 mL of fresh MHB and incubated with shaking (250 rpm) at 37°C. When cultures reached an absorbance of *A*_600__*nm*_ = 0.8, citral was added to a 2 or 4 mg/mL final concentration. Aliquots were removed at t_0__*h*_, t_1__*h*_, t_3__*h*_, and t_24__*h*_ post-exposure; the growth medium was then collected by centrifugation and filtration through two filters of 0.45 and 0.2 μm pore size, respectively. An organic extraction was repeated three times using 1 mL dichloromethane (for a total volume of 3 mL). The organic fractions were pooled and dried overnight in a chemical hood, and were finally re-dissolved in 500 μL of dichloromethane. Organic fractions and standards were diluted (1:200 for organic fraction and 1:2,000 for standard) in methanol. Ten microliter volumes were then sprayed as 8 mm bands on a TLC plate (Alugram^®^ Xtra SIL G UV254, Macherey-Nagel) using an automatic sampler (ATS4, Camag, Moirans, France) connected to visionCATS Camag TLC software V2.4. The TLC plates were developed in an automatic developing chamber (ADC 2, Camag) with a mobile phase containing cyclohexane:ethyl acetate (7:2) over a 70 mm migration distance. Spots were observed using UV-light at 254 nm (CV-415.LS, Uvitech, England).

### Measurement of Bacterial Surface Charge

Three independent mid-phase cultures of strain PA14 (ca. 10^7^ CFU/mL) were centrifuged (5,000 *g*, 10 min). The pellets were washed three times in distilled water and resuspended to yield 10^9^ CFU/mL prior to adding 8 mg/mL citral and antibiotics (tobramycin or colistin). Zeta potential was measured in a folded capillary cell (DTS 1070; Malvern Instruments, Worchestershire, United Kingdom) at 25°C using a Zetasizer Nano ZS (Malvern Instruments) equipped with a 633 nm He-Ne laser, and piloted with Zetasizer software v7.02. Bacteria were allowed to equilibrate for 120 s at 25°C. Zeta potential measurements were performed three times for each replicate sample. The Smoluchowski equation was used to calculate the potential from nine electrophoretic mobility determinations.

### Liquid Chromatography-High Resolution Mass Spectrometry (LC-HRMS)

Electrospray high-resolution mass spectrometry experiments (ESI-HRMS) were performed on an Agilent Accurate Mass QToF 6520 quadrupole time-of-flight instrument (Agilent Technologies, Les Ulis, France), hyphenated with a liquid chromatography system (Agilent 1200 HPLC system). Samples were prepared in methanol and 0.8 μL was injected. A Zorbax SB Agilent C_18_-column (50 mm × 2.1 mm i.d.) with a particle size of 1.8 μm maintained at 40°C was used. The elution was performed using a 0.5 mL/min mobile phase gradient of 0.05% formic acid in water (A) and 0.05% formic acid in acetonitrile (B), programmed as follows (A:B): 98:2 (*t* = 0 min), 0:100 (*t* = 8 min), 0:100 (*t* = 12.5 min), 98:2 (*t* = 12.6 min), 98:2 (*t* = 13 min). The acquisition of mass spectra was conducted in ESI (Electro-Spray Ionization) positive ionization mode using a capillary voltage of 4,000 V and the following conditions: nebulizer nitrogen gas pressure, 30 psig; drying gas flow rate, 8 L/min; and drying temperature, 340°C. The scan range was m/z 100–2,000 at 1 s/scan. Data acquisition was performed using MassHunter Qualitative Analysis software (B.07.00, Agilent).

### Raman Spectroscopy

The Raman spectroscopy experimental set up has previously been described (Foley and Enescu, 2007). It includes a Spectra Physics Nd/YAG laser model LAB-170-10 which delivers pulses with a duration of 5 ns at a repetition rate of 10 Hz. The equivalent power density at the sample was 30 mW mm^–2^ for a beam diameter of 1 cm. From our experience, the advantage of using pulsed excitation in Raman spectroscopy is that the relative contribution of the sample fluorescence to the detected signal is reduced due to the saturation of the excitation (this saturation does not occur for the Raman scattered light). The spectra were recorded using the second harmonic emission wavelength (532 nm) of the laser. The scattered light was detected at 90° using a Princeton Instruments spectroscopy system, which includes an Acton Spectra Pro 2500i monochromator with maximum resolution of 0.035 nm and a PIMAX-1024-RB CCD camera. In the present configuration, the spectral resolution of the system was 7 cm^–1^ as determined by the FWHM of the Raman spectrum of N_2_ in air. The intensity of the laser was constantly monitored by measuring the intensity of the N_2_ Raman band as obtained from the laser beam scattering on air. All spectra were then corrected with respect to the changes in laser intensity by dividing them by the intensity of the N_2_ Raman band acquisitioned over 400 laser shots. This was equivalent to a “normalization” of the measured intensities with respect to the Raman band of N_2_ in air. Consequently, the intensities of the different experimental Raman spectra reported in the present paper were measured in the same (arbitrary) unit, and can be directly compared. The study of the interaction between citral and antibiotics (tobramycin, colistin, or ticarcillin) was initiated by the rapid mixing of the two solutions. The concentration of citral was 40 mg/mL whilst the concentration of antibiotics was either 40 or 20 μg/mL. The reaction was monitored for 6 h by taking the Raman spectra every hour. The citral-antibiotics solution was maintained at 37°C for the duration of the measurements. Each spectrum was averaged over 1000 laser shots and for each time section an average of three experiments was performed.

### Synthesis of Tobracitryl

All the solvents were purified according to reported procedures, and the reagents used were commercially available. Methanol, ethyl acetate and petroleum ether were purchased from Sigma-Aldrich, and used without further purification. Column chromatography was performed on Merck silica gel (70–230 mesh). ^1^H NMR and ^13^C NMR spectra were recorded in CD_3_OD on a Bruker AC 300 spectrometer working at 300 and 75 MHz, respectively (the usual abbreviations are used: s: singlet, d: doublet, t: triplet, q: quadruplet, m: multiplet). All chemical shifts are given in ppm. The purity of the compounds was checked by analytical HPLC (C18 column, eluent CH_3_CN-water-TFA (90:10:0.025, v/v/v), 0.5-1 mL/min) with PDA detector spanning from 210 to 310 nm. All compounds possessed purity above 95%, as determined by analytical HPLC-PDA at 210 nm.

### General Procedure for the Synthesis of Tobracitryl 3

A mixture of citral (65 mg, 0.42 mmol) and tobramycin 200 mg (0.42 mmol) in absolute methanol (5 mL) was stirred at room temperature for 12 h. A small precipitate appeared and was eliminated by centrifugation. The supernatant was concentrated under vacuum leading to a yellow powder (150 mg) corresponding to the expected product **3** as a mixture of isomers in 58% yield. ^1^H NMR (CD_3_OD) mixture of isomers: *δ* = 8.20–8.40 (m, 2H), 5.96–6.08 (m, 2H), 5.06-5.20 (m, 4H), 3.96–4.01 (m, 1H), 3.86–3.90 (m, 1H), 3.62–3.76 (m, 4H), 3.49–3.57 (m, 2H), 3.37–3.44 (m, 2H), 3.17–3.29 (m, 3H), 3.02–3.07 (m, 1H), 2.81–2.91 (m, 3H), 2.17–2.26 (m, 7H), 1.92–2.05 (m, 6H), 1.68 (s, 7H), 1.64 (s, 7H). ^13^C (CD_3_OD) mixture of isomers: *δ* = 164.84, 164.43, 154.99, 154.53, 154.29, 133.39, 133.04, 132.96, 125.51, 124.73, 124.27, 124.22, 124.16, 124.11, 124.06, 101.18, 100.98, 90.63, 90.47, 77.11, 76.23, 75.87, 75.69, 74.40, 73.90, 73.63, 73.36, 71.47, 71.18, 69.93, 69.10, 67.84, 67.20, 62.51, 56.16, 51.53, 51.17, 50.88, 50.74, 43.50, 42.79, 41.15, 41.07, 37.58, 37.22, 36.85, 33.42, 33.28, 27.85, 27.74, 27.60, 27.01, 26.96, 26.93, 25.75, 25.70, 25.66, 24.48, 17.69, 17.65, 17.58, 17.56, 17.36, 17.32, 17.13, 17.06.

### General Procedure for the Synthesis of Reduced Tobracitryl 4

A mixture of tobracitryl **3** (50 mg, 8.1 10^–5^ mol) and sodium borohydride (31 mg, 8.1 10^–5^ mmol) in absolute methanol (5 mL) was stirred at room temperature for 12 h. The reaction was then quenched by adding water (1 mL). Stirring was maintained at room temperature for 20 min. After filtration over a pad of Celite washing with methanol and ethylacetate, the solvents were removed under vacuum and the crude mixture was purified by flash chromatography on Silicagel by using successively petroleum ether, ethylacetate and methanol affording the expected product **4** in 47% yield as a mixture of isomers. ^1^H NMR (MeOD): *δ* = 5.24–5.35 (m, 1H), 5.07–5.14 (m, 2H), 3.78–3.89 (m, 1H), 3.63–3.67 (m, 1H), 3.50–3.54 (m, 1H), 3.18–3.40 (m, 4H), 2.85–2.91 (m, 1H), 2.60–2.75 (m, 2H), 1.95–2.18 (m, 7H), 1.72–1.80 (m, 3H), 1.69 (s, 7H), 1.63 (s, 7H), 0.86–1.04 (m, 4H).^13^C (MeOD): *δ* = 170.30, 139.89, 139.52, 132.79, 132.69, 132.40, 125.16, 125.09, 124.05, 123.25, 123.04, 102.39, 88.22, 76.83, 74.78, 73.05, 70.12, 62.54, 56.53, 47.88, 45.05, 40.86, 40.78, 33.40, 33.14, 33.07, 31.21, 30.76, 27.75, 27.63, 26.06, 26.01, 25.95, 23.77, 17.91, 17.88, 17.84, 16.61, 16.46, 14.49.

## Data Availability Statement

The raw data supporting the conclusions of this article will be made available by the authors, without undue reservation.

## Author Contributions

CL, PP, GM, and CR conceived the study. AT, SF, GM, J-MB, EO, FT, AZ, BC, and YP carried out the experiments. CL, AT, and PP co-wrote the manuscript. CL did funding acquisition. All authors discussed the results and comments on the manuscript.

## Conflict of Interest

The authors declare that the research was conducted in the absence of any commercial or financial relationships that could be construed as a potential conflict of interest.

## Footnotes

1 www.cff.org
