# Supplementary material for: Negative Impact of Citral on Susceptibility of Pseudomonas aeruginosa to Antibiotics
Source: Front Microbiol. 2021 Jul 5;12:709838. doi: 10.3389/fmicb.2021.709838 (PMC8287888; doi:10.3389/fmicb.2021.709838)
Supplement: Supplementary file 1 [file Data_Sheet_1.PDF]

Table S1: Primers used for RT-qPCR experiments.

Primer	Sequence (5'→ 3')	Tm (°C)	Source
mexB1	ATCCGCCAGACCATCGCCA	60	(Hocquet et al., 2006)
mexB2	CATCACCAGGAACACGAGGAGG	60	
mexY1A	TTACCTCCTCCAGCGGC	60	(Jeannot et al., 2005)
mexY1B	GTGAGGCGCGCGTTGTG	60	
mexC1	TGCGGATTTCACCCAGACC	60	(Dumas et al., 2006)
mexC2	GGCCAAGGTGCCTTTCTTCA	60	
mexE4	CCAGGACCAGCACGAACT	60	(Dumas et al., 2006)
mexE5	CGACAACGCCAAGGGCGAGTTCACC	60	
rpsL3	GCAACTATCAACCAGCTGGTG	60	(Dumas et al., 2006)
rpsl5	GCTGTGCTCTTGCAGGTTGTG	60	

Figure S1: Susceptibility of bacterial strains to citral. 5 µL volumes (4.44 mg) of citral were spotted on paper disks. These latter were subsequently deposited on the surface of Mueller-Hinton agar plates inoculated with various clinical (PA14, PAO1, LESB58) or environmental (1341, 1393, 1423) strains of *P. aeruginosa* (on grey background). The activity of citral can be compared to that on the Gram-positive bacterium *Staphylococcus aureus.*

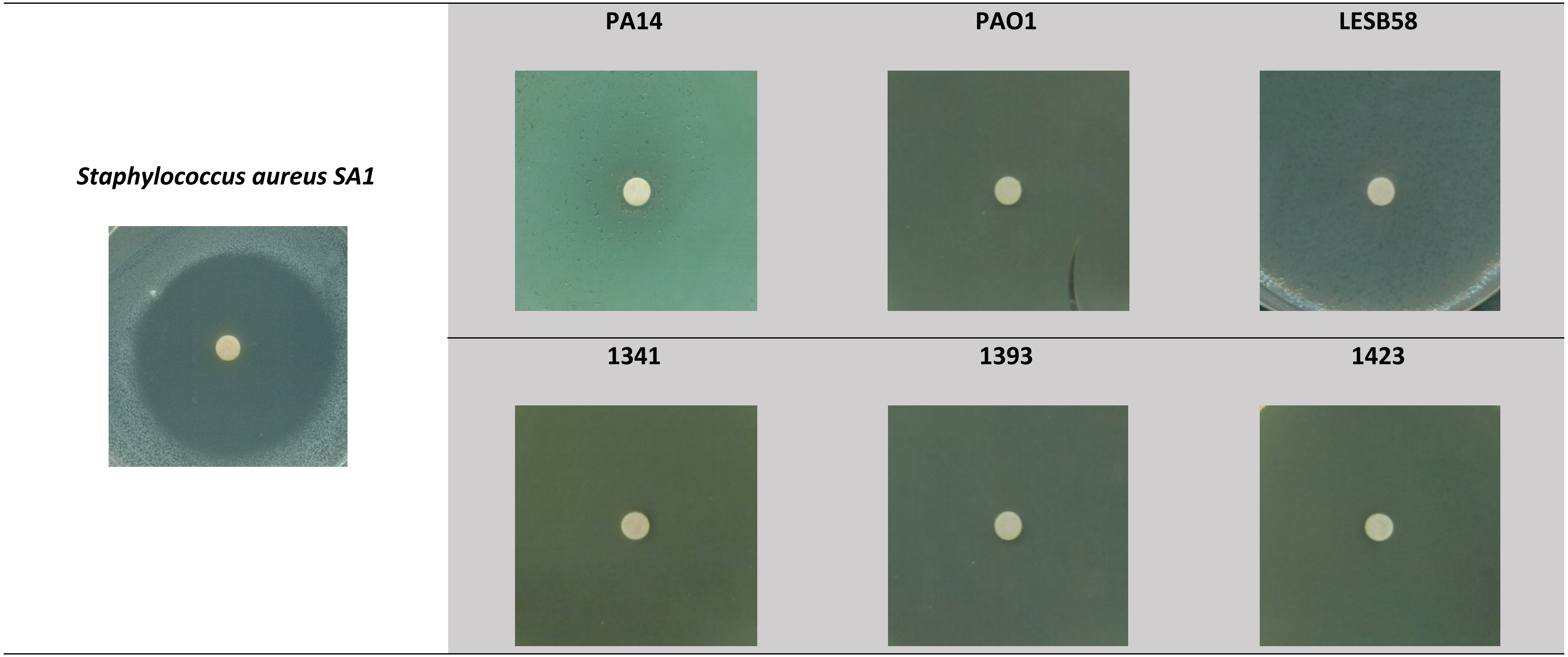

Figure S2. Antagonism between citral and aminoglycosides or colistin in different Gram-negative bacterial species.

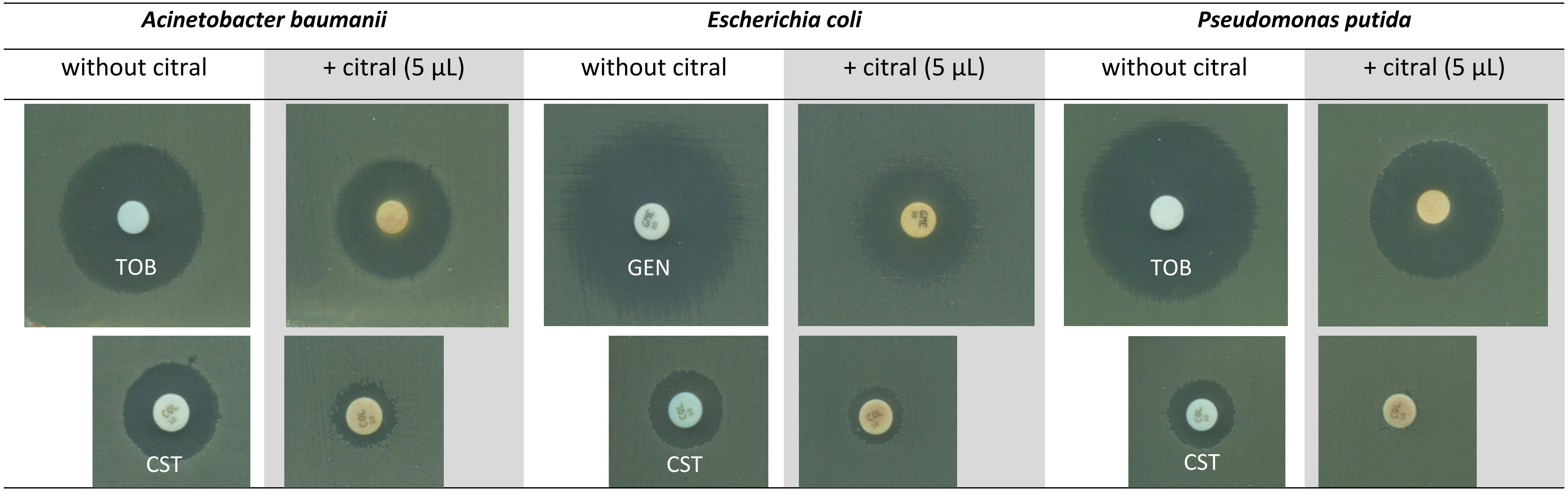

Addition of citral (5µl, i.e. 4.44 mg) to disks impregnated with tobramycin (TOB), gentamicin (GEN) or colistin (CST) causes a reduction of inhibition zones.

Figure S3. Raman spectrum of the citral C=O band. **(A)** The aldehyde band of citral progressively disappears when the product (40 mg/mL) is incubated with colistin (40 µg/mL) at 37°C for 360 min. **(B)** In contrast, no change is observed when citral is incubated with ticarcillin (40 µg/mL).

A

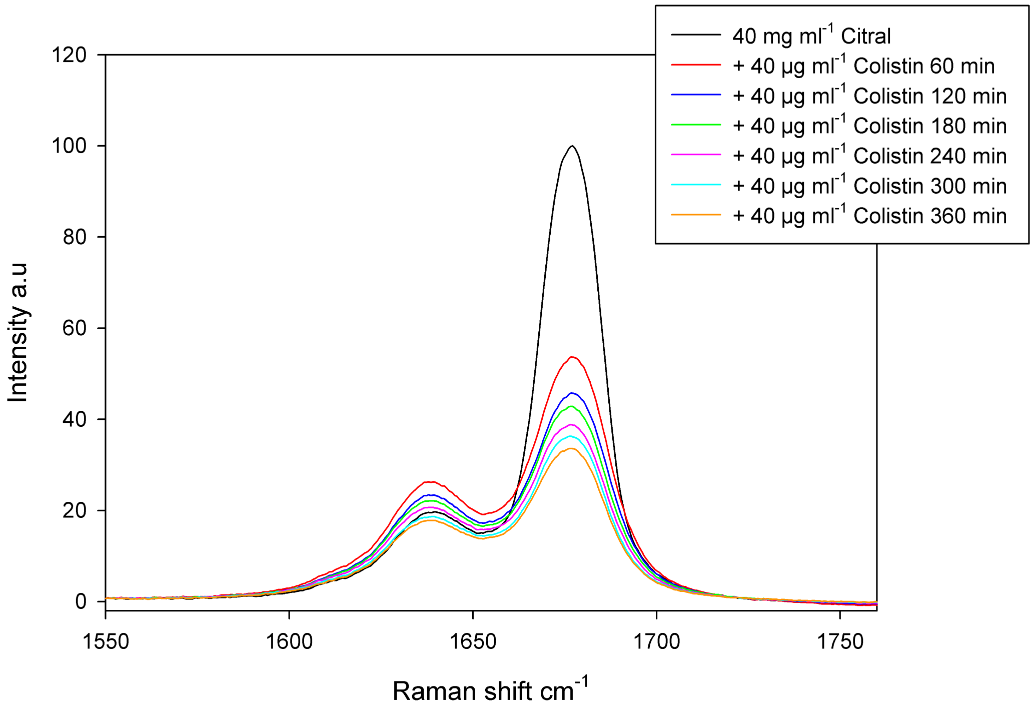

B

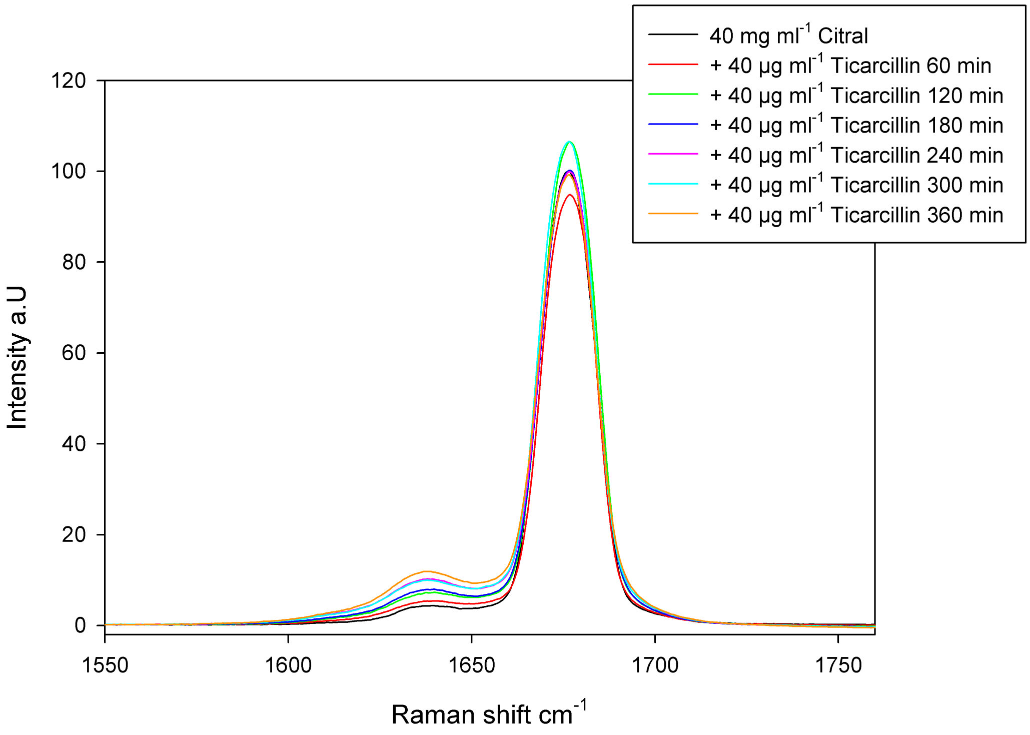

References

Dumas, J.L., Van Delden, C., Perron, K., and Köhler, T. (2006). Analysis of antibiotic resistance gene expression in *Pseudomonas aeruginosa* by quantitative real-time-PCR. *FEMS Microbiol Lett* 254**,** 217-225.

Hocquet, D., Nordmann, P., El Garch, F., Cabanne, L., and Plésiat, P. (2006). Involvement of the MexXY-OprM efflux system in emergence of cefepime resistance in clinical strains of *Pseudomonas aeruginosa*. *Antimicrob Agents Chemother* 50**,** 1347-1351.

Jeannot, K., Sobel, M.L., El Garch, F., Poole, K., and Plésiat, P. (2005). Induction of the MexXY efflux pump in *Pseudomonas aeruginosa* is dependent on drug-ribosome interaction. *J Bacteriol* 187**,** 5341-5346.
